# An Approach for Plant Leaf Image Segmentation Based on YOLOV8 and the Improved DEEPLABV3+

**DOI:** 10.3390/plants12193438

**Published:** 2023-09-29

**Authors:** Tingting Yang, Suyin Zhou, Aijun Xu, Junhua Ye, Jianxin Yin

**Affiliations:** 1 College of Chemistry and Materials Engineering, Zhejiang Agriculture and Forestry University, Hangzhou 311800, China; 15988822821@163.com; 2 Zhejiang Agriculture and Forestry University, Hangzhou 311800, China; zsy197733@163.com (S.Z.); yejunhua2020@zafu.edu.cn (J.Y.); 19970008@zafu.edu.cn (J.Y.); 3 Key Laboratory of State Forestry and Grassland Administration on Forestry Sensing Technology and Intelligent Equipment, Hangzhou 311300, China

**Keywords:** DenseASPP, YOLOv8, strip pooling, leaf, image segmentation

## Abstract

Accurate plant leaf image segmentation provides an effective basis for automatic leaf area estimation, species identification, and plant disease and pest monitoring. In this paper, based on our previous publicly available leaf dataset, an approach that fuses YOLOv8 and improved DeepLabv3+ is proposed for precise image segmentation of individual leaves. First, the leaf object detection algorithm-based YOLOv8 was introduced to reduce the interference of backgrounds on the second stage leaf segmentation task. Then, an improved DeepLabv3+ leaf segmentation method was proposed to more efficiently capture bar leaves and slender petioles. Densely connected atrous spatial pyramid pooling (DenseASPP) was used to replace the ASPP module, and the strip pooling (SP) strategy was simultaneously inserted, which enabled the backbone network to effectively capture long distance dependencies. The experimental results show that our proposed method, which combines YOLOv8 and the improved DeepLabv3+, achieves a 90.8% mean intersection over the union (mIoU) value for leaf segmentation on our public leaf dataset. When compared with the fully convolutional neural network (FCN), lite-reduced atrous spatial pyramid pooling (LR-ASPP), pyramid scene parsing network (PSPnet), U-Net, DeepLabv3, and DeepLabv3+, the proposed method improves the mIoU of leaves by 8.2, 8.4, 3.7, 4.6, 4.4, and 2.5 percentage points, respectively. Experimental results show that the performance of our method is significantly improved compared with the classical segmentation methods. The proposed method can thus effectively support the development of smart agroforestry.

## 1. Introduction

Image segmentation is a key aspect of image analysis and understanding in the field of computer vision. Plant leaf image segmentation is important for obtaining biomass characteristics [1]. The segmentation results can provide a basis for leaf area calculations [2], plant species identification [3], and plant disease and insect pest analysis [4]. Nondestructive leaf area monitoring is a crucial aspect for examining physiological features concerning plant growth [5]. It is an important parameter for evaluating the damage caused by leaf diseases and pests. To identify the plant species, knowing the leaf characteristics and structure is important. The morphological structures of leaves of different plant species may be the same, and different features are present in each leaf. Therefore, identifying features based on plant leaf edge are essential [6,7].

Traditional plant leaf segmentation methods mainly include the following: edge detection technique, threshold technique, region-based methods, clustering methods, and watershed methods. The edge detection techniques include the Sobel, Canny, Laplacian, and Fuzzy logic techniques [8]. These methods cannot obtain strong segmentation regions, and noise resistance and edge detection accuracy must be balanced. Threshold methods include the histogram bimodal method and OSTU [9,10], which demand few calculations and are simple to achieve. However, processing leaf images from outdoor conditions is difficult with these methods due to their poor performances on images with insignificant gray differences. The main forms of region-based methods are region growing, region splitting, and region merging [11,12]. These segmentation methods often cause excessive segmentation of images. The commonly used clustering-based segmentation methods include K-means and Fuzzy c-means, which do not require much data [13,14]. However, the results of these methods often depend on the initial parameter selections, and the models can easily fall into local optima, leading to low accuracy. Region- and watershed-based segmentation methods are sensitive to noise and perform poorly on segmentation tasks in complex environments [15]. The performance of these traditional methods cannot meet the requirements of our leaf images segmentation. Therefore, a segmentation algorithm with good performance is urgently needed.

The high precision and migration ability of deep learning techniques, such as FCN [16], U-Net [17], PSPNet [18], LR-ASPP [19], DeepLabv3 [20], and Mask-RCNN [21], have attracted an increasing number of researchers in the field of agriculture. The Mask R-CNN model has been used to segment leaf images with multiple targets and complicated backgrounds [3]. However, only 2000 images were used for segmentation experiments, and the data volume was insufficient. In [22], a study was presented that uses instance segmentation for the task of leaf and rust disease detection in apple orchards using Mask R-CNN. Background and blurred edges in the images make this very challenging. Although Mask R-CNN can detect both the target box and output instance segmentation mask, the detection box seemingly cannot improve segmentation accuracy. In [23], six convolutional neural network (CNN) architectures were trained for semantic segmentation of 766 images of individual leaves. Similarly, noise removal problems were present for complex image backgrounds. By employing a method based on the typical U-Net network and Xception architecture, Tamvakis et al. [1] achieved individual vine leaf segmentation and vein detection. However, low-contrast areas, e.g., leaf blades and vein endings, posed a problem because they shared similar pixel value intensities, causing the inferior lateral veins to be indistinguishable from the blades. Zhu et al. [4] used the two-stage LD-DeepLabv3+ model composed of Leaf-DeepLabv3+ and Disease-DeepLabv3+ for semantic segmentation of 4473 single apple leaf disease images; the proposed model could reduce the background impact. Lin et al. [24] presented a self-supervised leaf segmentation framework that is capable of segmenting leaf regions from the background under a complex illumination condition without annotated training data. However, the pixel-wise label assignment was updated and refined in an iterative manner, which may require hundreds of iterations to obtain sensible results. This paper [25] proposed an end-to-end semantic leaf segmentation model. The model used a deep convolutional neural network based on semantic segmentation. But, the background of the original images is very simple. The above studies on single-object segmentation of plant leaves exhibit some problems, such as few plant species, a small amount of data, a lack images of leaves in different states (e.g., curled or withered leaves), and a simple background.

Aiming to address existing problems, we chose to use a previously published public dataset that includes 9763 images of 39 plant species, including indoor and outdoor scenes. Our data amounts and number of species far exceed those of the previous related studies. The YOLOv8 model was proposed for extracting regions of interest and reducing the impact of the background on target segmentation. Then, an improved DeepLabv3+ [26] model was introduced to segment plant leaves. The main contributions of this study are as follows:

1. Plant leaf object detection and image segmentation datasets were constructed. The original images from a public leaf segmentation dataset [27] were annotated and transformed according to the YOLO dataset format to produce a new leaf dataset for object detection. The image segmentation task was performed on this public leaf dataset. The plant leaf dataset can be accessed at https://ytt917251944.github.io/dataset_jekyll (accessed on 24 November 2022).
2. To reduce the interference of the background in leaf segmentation tasks, an object detection algorithm based on YOLOv8 was proposed. The experimental results show that leaf object detection results significantly impact the second stage leaf segmentation task.
3. An improved DeepLabv3+ leaf segmentation method was proposed to more efficiently capture bar leaves and slender petioles. In this paper, DenseASPP was used to replace the ASPP module, and the SP strategy was simultaneously introduced, enabling the backbone network to effectively capture long-distance dependencies.
4. This new model, combined with the proposed YOLOv8 and improved DeepLabv3+, was designed to accurately segment individual leaves in various states, such as regular, curled or withered, and dorsal leaves, making the model more feasible for leaf characteristics collected in natural environments.

## 2. Results and Discussion

### 2.1. Training Environment and Evaluation Indicators

The experimental setup was as follows: Intel Core i7-12700KF CPU, 64 GB RAM, Nvidia GeForce RTX3090Ti GPU, 10 GB of video memory, CUDA Toolkit 9.0, CUDNN V11.7., Python 3.7.12, and the Ubuntu 22.04 operating system.

To quantitatively evaluate the performance of the proposed method and the compared methods, three metrics were used as the object detection evaluation criteria: precision, recall, and mean average precision (mAP). The metrics were calculated as follows (Equation (1)):

(1) $$\left\{\begin{array}{c} \mathrm{Precision}=\frac{TP}{TP+FP}\\ \mathrm{Recall}=\frac{TP}{TP+FN}\\ \mathrm{AP}=\int_{0}^{1}P_{i}(r_{i})d(r_{i}),\;\mathrm{mAP}=\frac{\sum_{i=1}^{N}\int_{0}^{1}P_{i}(r_{i})d(r_{i})}{N} \end{array}\right.$$

where TP and FP denote the numbers of correct and incorrect identifications, respectively. FN denotes the number of all true cases not detected by the network model. *N* is the number of plant leaf species. *P_i_* (*r_i_*) is the precision at recall *r_i_*_._

Two metrics were used as the segmentation evaluation criteria: mean intersection over union (mIoU) and mean pixel accuracy (mPA). The metrics were calculated as follows (Equation (2)):

(2) $$\left\{\begin{array}{c} \mathrm{IoU}=\frac{TP}{FN+FP+TP},\;\mathrm{mIoU}=\frac{1}{N+1}\sum_{i=0}^{N}\frac{TP}{FN+FP+TP}\\ \mathrm{PA}=\frac{TP+TN}{FN+TP+FP+TN},\;\mathrm{mPA}=\frac{1}{N+1}\sum_{i=0}^{N}\frac{TP+TN}{FN+TP+FP+TN} \end{array}\right.$$

where TN denotes the number of all false cases detected by the network model.

### 2.2. YOLOv8 Model Results and Analysis

**Figure 1:**
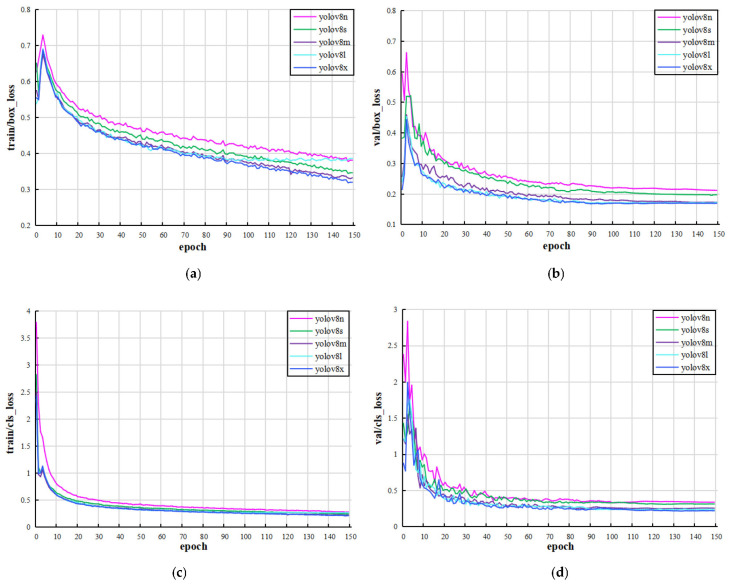
(**a**,**b**) are the training and validation loss function curves of the object detection frame, respectively. (**c**,**d**) are the training and validation loss function curves of plant leaf classification, respectively.

The YOLOv8 series consists of five models, namely YOLOv8n, YOLOv8s, YOLOv8m, YOLOv8l, and YOLOv8x. For leaf image object detection, the hyperparameters were set as epoch 150, batch size 32, initial learning rate 0.01, momentum 0.937, and weight attenuation coefficient 0.0005. Then, the trained model was evaluated by the validation and test sets. Figure 1 shows the loss function curves of the training and validation processing based on the YOLOv8 model. The loss function curves decrease steadily to flatness, indicating that the model converges.

**Figure 2:**
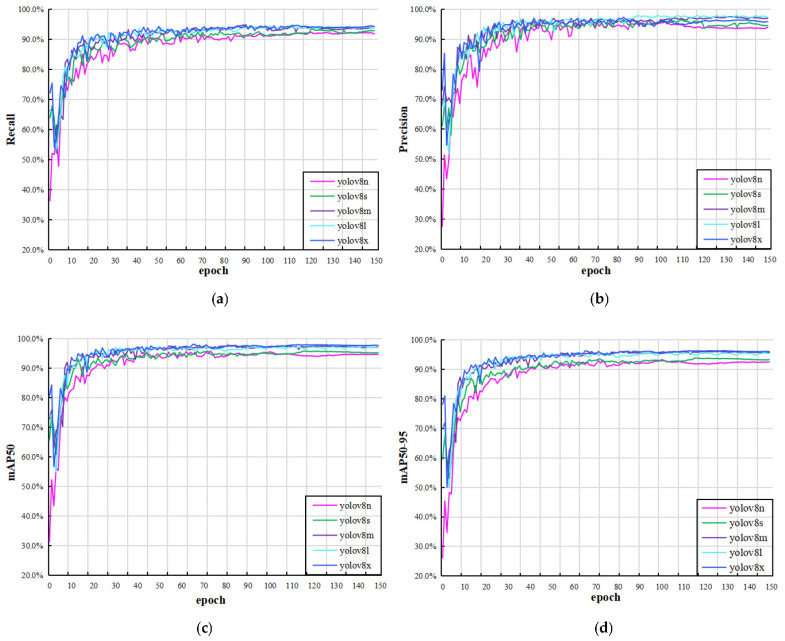
(**a**–**d**) are respectively the change curves of recall, precision, mAP50 and mAP50–95 values of the YOLOv8 series on the validation set.

Figure 2 shows the change curves of recall, precision, mAP50, and mAP50–95 of the validation set. These curves indicate that the network converges quickly and tends to be stable. In addition, YOLOv8n is the smallest and fastest model, and YOLOv8x is the most accurate but slowest model. In this paper, five models were trained and verified on our leaf dataset. The mAP^val^50 and mAP^val^50–95 values of YOLOv8m are much higher than those of YOLOv8n and YOLOv8s, and the index values of YOLOv8m are comparable to those of YOLOv8l and YOLOv8x; Table 1 depicts these details. Therefore, YOLOv8m was selected as the main network for the object detection task in this paper.

**Figure 3:**
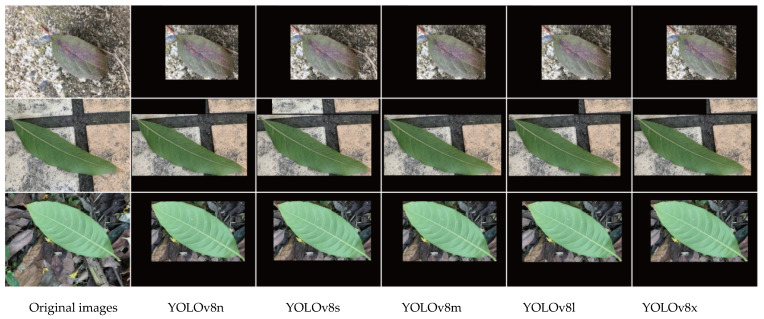
Example images of object detection results of YOLOv8 series models on the test set.

Figure 3 displays examples of the object detection results of the YOLOv8 series models on our test set. The prediction box deviations of the YOLOv8n and YOLOv8s models are large, while the prediction box deviations of the YOLOv8m, YOLOv8l, and YOLOv8x models are smaller than the ground truth box.

### 2.3. Results and Analysis of Different Segmentation Models

**Figure 4:**
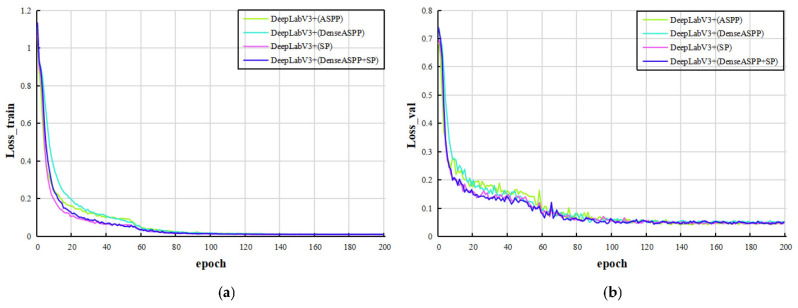

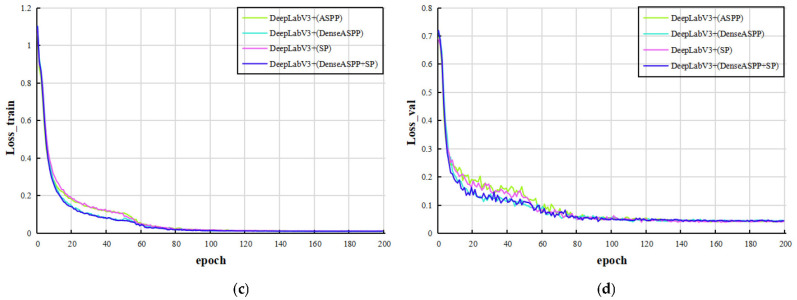
Image segmentation loss function change curves: (**a**,**b**) are the training and validation loss function curves based on the improved DeepLabv3+ leaf image segmentation, respectively. (**c**,**d**) are the training and validation loss function curves based on YOLOv8m and improved DeepLabv3+ leaf image segmentation, respectively.

In this paper, an approach for plant leaf image segmentation based on YOLOv8 and improved DeepLabv3+ was proposed. YOLOv8 was first used to carry out object detection on our leaf dataset, and the detection results were then input into the improved DeepLabv3+ model for segmentation. To better verify the segmentation performance of our proposed DeepLabv3+ (DenseASPP + SP) method, we conducted DeepLabv3+ (ASPP), DeepLabv3+ (DenseASPP), and DeepLabv3+ (SP) experiments. For the segmentation task, the hyperparameters were set as epoch 200, batch size 8, initial learning rate 0.01, momentum 0.9, and weight attenuation coefficient 0.0001. Then, the trained model was evaluated on the validation and test datasets. Figure 4 shows the segmentation loss function curves of different models on our leaf dataset. The loss function curves of different models decline steadily and flatten, indicating that the models converge.

To further validate the segmentation performance of the proposed method on our leaf dataset, the proposed method was compared with typical models, such as FCN, LR-ASPP, U-Net, and DeepLabv3. The specific comparison results are shown in Table 2. The overall performance of our proposed DeepLabv3+ (DenseASPP + SP) was better than that of other models. The segmentation results of the fused YOLOv8m model were also better than those of the other models.

The results of different segmentation models are shown in Table 2. The mIoUs of the classic segmentation models based on YOLOv8m, e.g., FCN, LR-ASPP, PSPnet, U-Net, and DeepLabv3, increased by 2.8, 3.3, 2.6, 1.9, and 3.4 percentage points, respectively, compared to models without YOLOv8m processing. Compared with the FCN, LR-ASPP, PSPnet, U-Net, and DeepLabv3 models, the mIoU of DeepLabv3+ (DenseASPP + SP) based on YOLOv8m increased by 8.2, 8.4, 3.7, 4.6, and 4.4 percentage points, respectively. The performance of our proposed model is significantly better than that of the classic image segmentation models.

Furthermore, introducing DenseASPP or SP networks cannot improve the segmentation performance of DeepLabv3+, but combining DenseASPP and SP networks can do so. Our DeepLabv3+ (DenseASPP + SP) model based on YOLOv8 is 1.8 percentage points higher than the mIoU of the model not based on YOLOv8. The mIoU and mPA values of our model based on YOLOv8 are 2.5 and 1.4 percentage points higher than those of the original DeepLabv3+ (ASPP) model. Table 3 shows the segmentation performance of different segmentation models on the test set. Although the value of each index is slightly lower than that of the validation set, these results still indicate that these models have better generalizability. Moreover, Table 3 demonstrates that the fused YOLOv8 and improved DeepLabv3+ model still has the best segmentation performance on the test set.

To some extent, the overall indicator values cannot directly reflect the segmentation results of each species. Therefore, the segmentation results of the proposed method on the validation and test sets are analyzed from the perspective of each species to evaluate the performance of the model.

**Figure 5:**
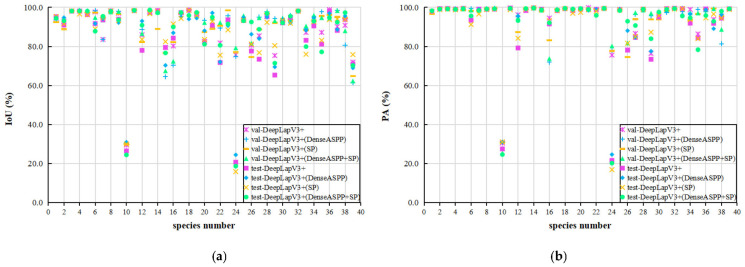
Segmentation results of different segmentation methods based on YOLOv8 on the validation and test sets. (**a**,**b**) are the values of IoU and PA. The horizontal axis represents the species number.

**Figure 6:**
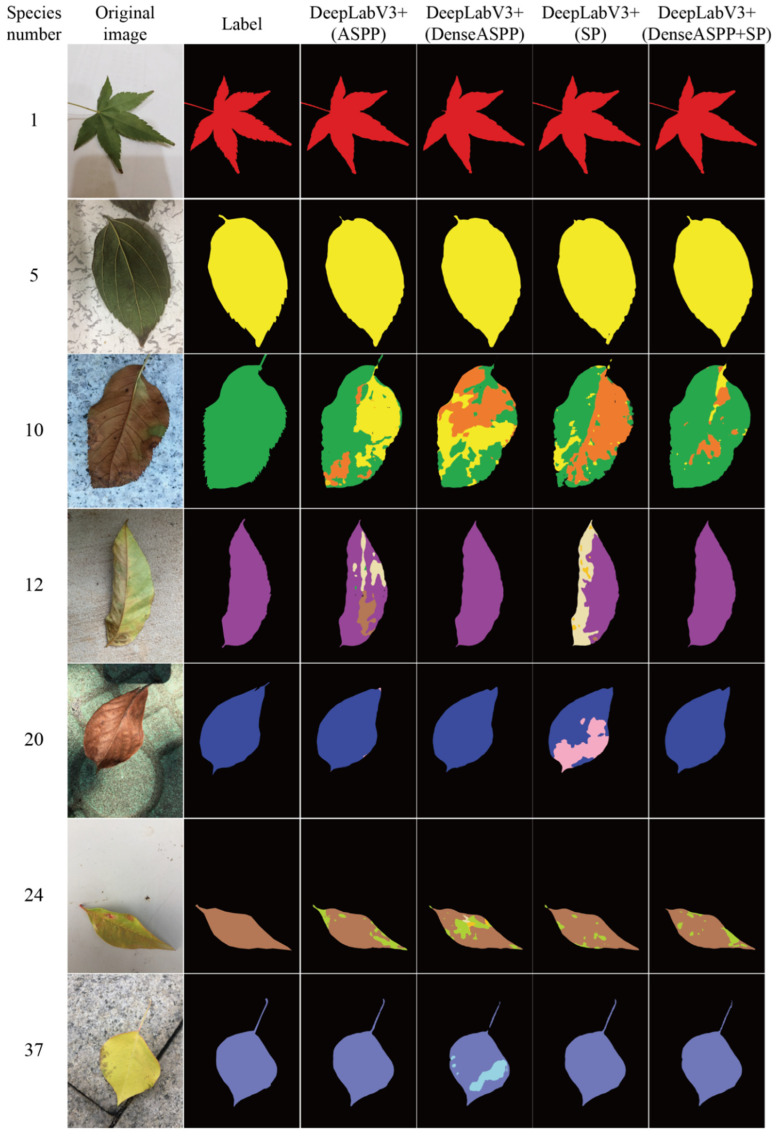
Example segmentation results of different segmentation methods based on YOLOv8 on the test set.

Figure 5 depicts the segmentation results of different segmentation methods based on YOLOv8 on the verification and test sets. Comparison revealed that the segmentation performance of leaf species number 24 was different on the verification and test sets. This is because the images from the test set were mostly withered leaves with spots (see Figure 6, species number 24); that is, there may have been fewer spotted or withered leaf images of this species in the training and validation sets. For leaf species number 10, the segmentation models performed poorly on both the validation and test sets. The analysis results showed that these images contained many uneven spotted leaves. Our DeepLabv3+ (DenseASPP + SP) model can regionally segment such leaf images but the classification effect is poor, resulting in segmentation results as shown in Figure 6 (species number 10). Observations of species numbers 1, 5, and 37 reveal that our proposed method can effectively segment the petioles of leaves. In addition, the segmentation results of leaves from other species on validation and test sets show that the proposed segmentation method is effective on our leaf dataset. Although the IoU and PA values of the test set are slightly lower than those of the validation set, the DeepLabv3+ (DenseASPP + SP) model still has good generalizability.

### 2.4. Discussion

In this subsection, these classical segmentation networks, such as FCN, U-Net, PSPNet, LR-ASPP, and DeepLabv3, are discussed. Then, the network structures of DeepLabv3+ (ASPP), DeepLabv3+ (DenseASPP), DeepLabv3+ (SP), and DeepLabv3+ (DenseASPP + SP) are discussed. Therefore, the effectiveness of our DeepLabv3+ (DenseASPP + SP) is demonstrated by experiment results. 

The results of the different classical segmentation network structures on the leaf validation set are displayed in Table 2. The performance of the original DeepLabv3+ [26] model is slightly better than these models in the literature [16,17,18,19,20]. The mIOU values of these models based on the YOLOv8 network all remarkably improved, which indicates that the object detection algorithm is effective in removing image background. Our method, replacing the ASPP module with the DenseASPP module [28] and introducing the SP module [29], significantly increases the segmentation accuracy of leaves. Compared with the FCN, LR-ASPP, PSPnet, U-Net, and DeepLabv3 models, the mIoU of our method based on YOLOv8 increased by 8.2, 8.4, 3.7, 4.6, and 4.4 percentage points, respectively. This is because the multiscale features extracted by the DenseASPP module have higher discriminability than those extracted by the ASPP module, which facilitates the network to segment the target leaf from the complex environment. In addition, introducing the SP module can improve the accuracy of leaf segmentation by capturing bar leaves and slender petioles. 

Then, the images and leaf labels in the test set were used to calculate the evaluation indicators of various network structures. The results of the different modified structures on the leaf test set are displayed in Table 3. The modified DenseASPP and the SP module based on YOLOv8 can, respectively, promote the segmentation accuracy of the leaf. They increase the IoU of leaf segmentation by 2.4 and 1.4 percentage points, respectively. The reason is that the modified DenseASPP model uses different dilated rates, which reduce the loss of local information. The accuracy of our method based on YOLOv8 in the test set reaches 88.1% in mIoU and 93.0% in mPA, which is 2.6 and 1.4 percentage points higher than that of the original DeepLabv3+ (ASPP), respectively. The results significantly demonstrate the effectiveness of our proposed method for leaf segmentation. 

The example images depicted in Figure 6 are from the test set and mainly consist of curled, withered, spotted, long petioles, and reverse leaves. The leaf of species number 1 has a long petiole and toothed edge, and the petiole cannot be completely segmented by the DeepLabv3+ (DenseASPP) model. Although the proposed method can effectively segment the petiole, there is still room for improving the refined edge segmentation of leaves. Species numbers 10, 12, and 24 all have uneven spots on their leaves. For uniformly withered leaf images (see Figure 6, species number 20), the DeepLabv3+ (DenseASPP + SP) model can completely segment the leaf. However, if tender or withered areas are present in a single leaf (as in species number 10), our model can segment the entire leaf more completely than other models, but classification errors are present. Improving the performance of the classifier in the segmentation task would be meaningful, so this is our next research focus. 

## 3. Materials and Methods

### 3.1. Leaf Dataset

#### 3.1.1. Image Acquisition

Many of the most advanced models require a vast amount of labeled data to obtain strong results. Therefore, collecting sufficient training images is essential. In our previous research, we published an open-source dataset of plant leaf images with indoor and outdoor backgrounds [27]. These plant leaf images were photographed using different devices, such as mobile phones, iPads, and cameras. These images were collected from Nanjing, Changzhou, Linyi, and Hangzhou urban streets and covered 39 common urban plant species. The images were collected from February to October 2022.

**Figure 7:**
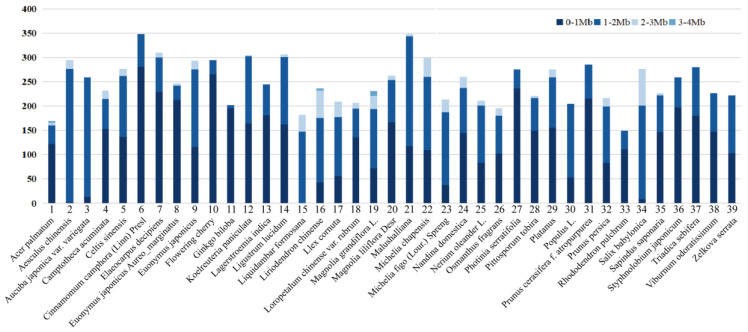
Leaf image size distribution of different plant species. The horizontal axis represents the species number and name in this paper. The vertical axis represents the number of leaf images for each species. The specific species numbers in Figure 5 correspond to those plant species in Figure 7.

- Our dataset was collected in natural environments. We, respectively, captured images of the same plant species on different city streets and under different lighting intensities, object scales, growth stages, and shooting perspectives to increase the diversity of our dataset.
- We adopted various data augmentation techniques, such as cropping, mirroring, and rotating, to increase the magnitude of our dataset; we then performed image compression processing. A total of 9763 plant leaf images were acquired. The number of different plant species acquired is shown in Figure 7. The leaf image size was distributed from approximately 0 to 2 Mb; this range accounts for 94.7% of the total.

#### 3.1.2. Image Annotation

**Figure 8:**
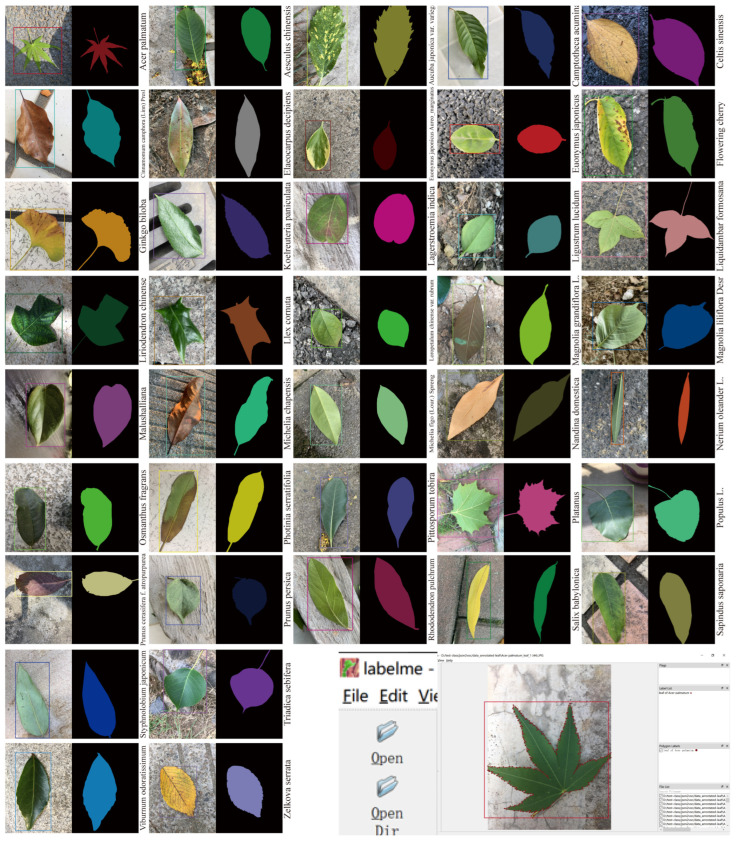
Example annotation images of plant leaf object detection and instance segmentation. The LabelMe tool receives multiple .json files. Different color rectangular boxes represent object detection boxes of different plant species. Different color squares represent segmentation labels of different plant species.

The leaf images were annotated by a team of 10 professional image annotators. Seven of the annotators performed initial quality assurance, and three verified the annotations. During the object detection task, a rectangular box was used to label the leaf images. For the instance of segmentation task, fine annotations were provided at the pixel level, as shown in Figure 8. The images were annotated by polygons, with each polygon consisting of 20–200 points (LabelMe).

The leaf dataset was then divided into training, validation, and test sets at a ratio of 8:1:1. Table 4 provides the details of these sets. The object detection and segmentation dataset formats follow YOLO and VOC2012 [30], respectively.

### 3.2. Methods

**Figure 9:**
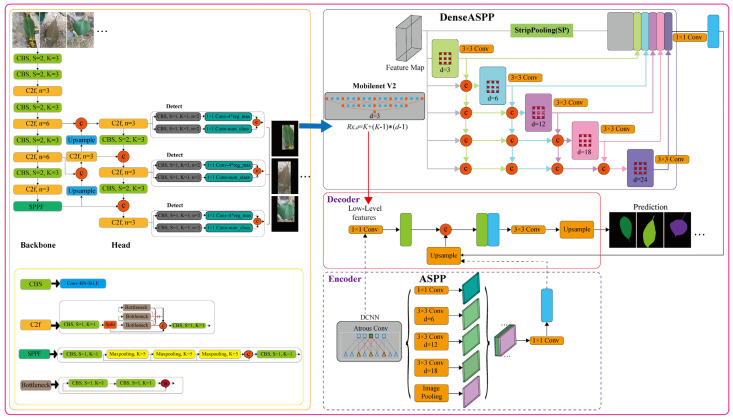
The network structure based on YOLOv8 and improved DeepLabv3+ for plant leaf segmentation, where *S* is the step size, *n* is the number of bottlenecks, *K* is the size of the convolution kernel, *d* is the dilation rate, and *R_K_*_,*d*_ is the receptive field.

To reduce the impact of image background on leaf segmentation, the YOLOv8 network was proposed for obtaining the regions of interest in the leaf images. On this basis, the improved DeepLabv3+ model was used for leaf image segmentation. The specific leaf object detection and segmentation network is depicted in Figure 9.

#### 3.2.1. YOLOv8 Network

YOLO is an object detection system that makes predictions based on global image information. Since Joseph Redmon, Ali Farhadi, and others [31] proposed the original model, researchers in the field have proposed multiple updates and iterations of YOLO [32,33]. As a result, the model performance has become increasingly powerful.

**Figure 10:**
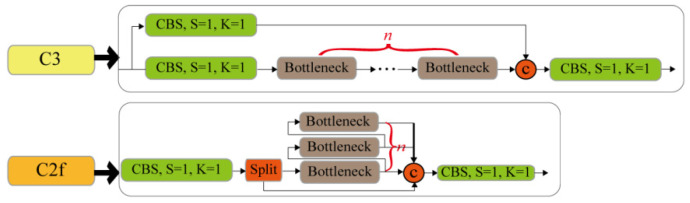
YOLOv5 C3 structure and YOLOv8 C2f structure.

YOLOv8 builds on previous YOLO releases, introducing new features and improvements to further enhance performance and flexibility. Its core features and modifications can be summarized as follows:

(1) A new state-of-the-art model is provided. Similar to YOLOv5, different size models of the n/s/m/l/x scale are also provided based on the scaling coefficient to meet the requirements of different scenarios.
(2) The backbone network and neck part may refer to the design concept of YOLOv7 ELAN, replacing the C3 structure of YOLOv5 with the C2f structure, which exhibits more abundant gradient flow and adjusts different channel numbers for different scale models, which significantly improves model performance, as shown in Figure 10.
(3) Compared with YOLOv5, the head part is changed to the current mainstream decoupling head structure, separating the classification and detection heads and changing the model from anchor-based to anchor-free. The anchor-free model abandons or bypasses the anchor concept, uses a streamlined method to determine positive and negative samples, simultaneously reaches or even exceeds the accuracy of the anchor-based model, and has a faster speed than the anchor-based model.
(4) The TaskAlignedAssignor positive sample allocation strategy is adopted for loss function calculation. The classification loss is the VariFocal Loss (VFL), and the regression loss is the Complete-IoU (CIoU) Loss + Distribution Focal Loss (DFL).

The TaskAlignedAssignor uses the matching strategy of selecting positive samples based on weighted classification and regression scores. This is calculated as follows (Equation (3)):

(3) $$t=s^{\mu }\times u^{\beta }$$

where *s* is the prediction score corresponding to the annotated category. *u* is the IoU of the prediction box and the ground-truth box. *μ* and *β* are the weight hyperparameters. When *μ* and *β* are multiplied together, the degree of alignment can be measured.

**VFL** [34] primarily considers asymmetric weighting operations. **VFL** is calculated as follows (Equation (4)):

(4) $$VFL(p,q)=\left\{\begin{array}{c} -q(q\log(p)+(1-q)\log(1-p))\;q>0\\ -\omega p^{\gamma }\log(1-p)\;\;\;\;\;\;\;q=0 \end{array}\right.$$

where ω and γ are hyperparameters, *p* is the predicted IoU-aware classification score, *p* ∈ [0, 1], and *q* is the label. When the sample is positive, *q* is the IoU between the predicted and ground-truth values. When the sample is negative, *q* is equal to 0.

*CIoU*, which is based on *IoU*, considers the constraints of the central point distance and aspect ratio. *CIoU* is calculated as follows (Equation (5)):

(5) $$\left\{\begin{array}{c} L_{CIoU}=1-IoU+\frac{\rho^{2}(b,b^{gt})}{c^{2}}+\alpha v\\ v=\frac{4}{\pi^{2}}{\left(arctan\frac{w^{gt}}{h^{gt}}-arctan\frac{w^{p}}{h^{p}}\right)}^{2}\\ \alpha =\frac{v}{(1-IoU)+v} \end{array}\right.$$

where *b* and *b^gt^* represent the center points of the prediction box and ground-truth box, respectively. ρ represents the Euclidean distance between the two center points. *c* represents the diagonal distance of the smallest closed area that can contain the prediction box and the ground-truth box simultaneously. *α* and *v* comprise the impact factor. *w^gt^* and *h^gt^* are the width and height of the ground-truth frame, and *w^p^* and *h^p^* are the width and height of the prediction frame, respectively.

**DFL** [35] primarily models the position of the box as a general distribution and optimizes the probability of the two positions closest to the label *y* in the form of cross-entropy. This allows the network to more quickly focus on the distribution of adjacent regions of the target position and obtain the weight of the integer coordinates around the distance by linear interpolation. **DFL** is calculated as follows (Equation (6)):

(6) $$DFL(S_{i},S_{i+1})=-((y_{i+1}-y)log(S_{i})+(y{-y}_{i})log(S_{i+1}))$$

The probabilities of the two predicted values *y_i_* and *y_i_*_+*1*_ near label y are *S_i_* and *S_i_*_+1_.

#### 3.2.2. Improved DeepLabv3+

DeepLabv3+ is a network architecture based on the DeepLab series, which is a collection of atrous convolutional and multiscale series models. DeepLabv3+ applies depthwise separable convolution to the ASPP and decoder modules. ASPP obtains multiple receptive fields of different sizes by using different pooling kernels to acquire multiscale context information. The decoder module captures clear target boundary information by gradually restoring space, thus producing a faster and stronger encoder–decoder network. The details of the ASPP and decoder modules are depicted in Figure 9.

Although ASPP can generate multiscale features, the generated features are not sufficiently dense. Based on the ASPP and DenseNet networks [28], DenseASPP expands the convolution layers in a cascading manner, with the dilation rate increasing layer by layer. The layer with the smallest dilation rate is placed at the bottom, while the layer with the largest dilation rate is placed at the top. The output and input feature maps of each atrous layer are connected to all outputs from lower layers, and the connected feature maps are then fed to the next layer. The final output of DenseASPP is a feature map generated by the multidilation rate and multiscale atrous convolution. This structure can form a denser and larger feature pyramid with only a small number of expanded convolutional layers.

The ASPP model formula is as follows (Equation (7)):

(7) $$y=H_{3,6}(x)+H_{3,12}(x){+H}_{3,18}(x)+H_{3,24}(x)$$

where *H_K_*_,*d*_(*x*) represents an atrous convolution and *y* represents fusion features.

Each atrous layer in DenseASPP can be represented as follows (Equation (8)):

(8) $$y_{l}=H_{K,d_{l}}([y_{l-1},y_{l-2},\ldots ,y_{0}])$$

where *d_l_* represents the dilation rate of layer *l*. […] represents the cascading concatenation operation. [*y_l_*_−1_, *y_l_*_−2_, …, *y*_0_] represents the feature generated after concatenating the output of all layers before the current layer. *y_l_* is the feature map received by the current layer.

Thus, DenseASPP needs to connect only four convolution layers with different dilation rates [3,6,12,18], using the DenseNet connection to form a dense feature pyramid. Each layer merges multiple different scale features of the previous layers in parallel; that is, each layer generates fusion features of multiscale large receptive fields.

Assuming that the dilation rates *d* are [6,12,18,24], the maximum receptive field *R_ASPP_* is (Equation (9)):

(9) $$R_{ASPP}=max[R_{3,6},R_{3,12},R_{3,18},R_{3,24}]=R_{3,24}$$

The maximum receptive field *R_DenseASPP_* is (Equation (10)):

(10) $$R_{DenseASPP}=R_{3,6}+R_{3,12}+R_{3,18}+R_{3,24}-3$$

**Figure 11:**
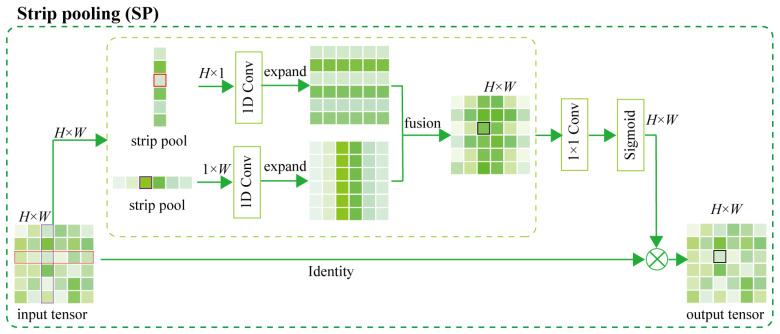
Strip pooling (SP) structure.

Additionally, to more effectively capture bar leaves and slender petioles in the leaf dataset, the SP [29] strategy is introduced in this paper. This strategy enables the backbone network to effectively capture long-distance dependency, as shown in Figure 11.

The following is assumed: The 2D tensor is *x*, the size is *H* × *W*, the pooling window is (*H*, 1) or (1, *W*) and the pooling space range is *h* × *w*. Unlike 2D average pooling, SP averages all feature values in a row or column. The horizontal *h* and vertical *v* pooling expressions are (Equation (11)):

(11) $$y_{i}^{h}=\frac{1}{W}\sum_{0\leq j\leq W}x_{i,j\;},{\;y}_{j}^{v}=\frac{1}{H}\sum_{0\leq i\leq H}x_{i,j}$$

Suppose the input size is *C* × *H* × *W*, and *C* is the number of input channels. The tensor *x* is first input into two parallel paths, each of which contains a horizontal (1, *W*) or vertical (*H*, 1) strip pooling layer. The 1D convolution layer of *K* = 3 is then used to expand the two output feature maps, ensuring that the dimensions of the feature maps of the two branches after expansion are consistent. Next, the corresponding positions of the two feature maps are summed, as shown in Figure 11. Suppose $y_{c,i}^{h}\in R^{C\times W}$ and $y_{c,j}^{v}\in R^{C\times H}$; then, $y\in R^{C\times H\times W}$ can be expressed as (Equation (12)):

(12) $$y_{c,i,j}=y_{c,i}^{h}+y_{c,j}^{v}$$

The final result is obtained by point multiplication of the sigmoid function and the original feature map. This is calculated as follows (Equation (13)):

(13) z=Scale(x,σ(f(y))

where *Scale* (.,.) represents the bitwise multiplication of elements, *σ* represents the sigmoid function, and *f* represents the 1 × 1 convolution.

## 4. Conclusions

In this work, the leaf object detection algorithm-based YOLOv8m was used to reduce the interference of background in the leaf segmentation task. Then, an improved DeepLabv3+ leaf segmentation method was proposed to more efficiently capture bar leaves and slender petioles. DenseASPP was used to replace the ASPP module, and the SP strategy was simultaneously introduced, enabling the backbone network to effectively capture long-distance dependencies. Combined with the proposed YOLOv8 and improved DeepLabv3+, the new model was designed to effectively reduce the influence of complex backgrounds and significantly improve the accuracy of the segmentation results. The experimental results show that the proposed method achieves 90.8% mIoU on the validation set and 88.1% mIoU on the test set. Our approach exhibits significantly higher accuracy than other models. Therefore, the proposed method achieves the accurate segmentation of single plant leaves in different states, providing support for calculating leaf area, identifying plant species, and obtaining information about plant diseases and insect pests. In subsequent studies, the model will be optimized for spotted leaf segmentation to make it more suitable for practical applications.

## Figures and Tables

**Table 1:** Different models of the YOLOv8 series.

Model	Size (Pixels)	Params (M)	FLOPs (B)	Layers	Recall	Precision	mAP^val^ 50	mAP^val^ 50–95
YOLOv8n	640	3.0	8.2	225	90.9	94.7	95.3	92.7
YOLOv8s	640	11.2	28.7	225	91.2	96.4	95.7	93.0
YOLOv8m	640	25.9	79.2	295	93.2	97.4	97.7	95.9
YOLOv8l	640	43.7	165.6	365	93.8	97.8	97.6	95.8
YOLOv8x	640	68.2	258.3	365	93.9	97.3	98.0	96.2

**Table 2:** Comparison of segmentation performance of different models on the validation set.

	Not Based on YOLOv8m		Based on YOLOv8m	
Model	mIoU	mPA	mIoU	mPA
FCN (resnet50) [16]	82.6	-	85.4	-
LR-ASPP [19]	82.4	-	85.7	-
PSPnet (mobilenet) [18]	87.1	-	89.7	-
U-Net (resnet50) [17]	86.2	93.0	88.1	93.9
DeepLabv3 [20]	86.4	-	89.8	-
DeepLabv3+ (ASPP) [26]	88.3	93.9	89.6	94.6
DeepLabv3+ (DenseASPP) [28]	87.8	93.7	89.5	94.5
DeepLabv3+ (SP) [29]	88.4	94.0	89.9	94.7
Ours: DeepLabv3+ (DenseASPP + SP)	89.0	94.3	90.8	95.3

**Table 3:** Comparison of the segmentation performance of different models on the test set.

	Not Based on YOLOv8m		Based on YOLOv8m	
Model	mIoU	mPA	mIoU	mPA
DeepLabv3+ (ASPP)	85.5	91.6	86.7	92.1
DeepLabv3+ (DenseASPP)	86.0	91.8	87.9	92.9
DeepLabv3+ (SP)	86.1	92.0	87.4	92.7
Ours: DeepLabv3+ (DenseASPP + SP)	86.7	92.3	88.1	93.0

**Table 4:** Leaf object detection and segmentation dataset construction.

Leaf Dataset	Ratio	Number	Dataset Format (Object Detection)	Dataset Format (Segmentation)
training set	8	7813	YOLO	VOC2012
validation set	1	975		
test set	1	975		

## Data Availability

The plant leaf dataset can be accessed at https://ytt917251944.github.io/dataset_jekyll (accessed on 24 November 2022).
